# High-quality genome assembly and annotation of *Porodaedalea mongolica* and *Porodaedalea schrenkiana* provide insights into potential industrial and medical application

**DOI:** 10.1093/g3journal/jkaf195

**Published:** 2025-09-25

**Authors:** Shu-Bin Liu, Heng Zhao, Fang Wu

**Affiliations:** School of Ecology and Nature Conservation, Beijing Forestry University, Beijing 100083, China; School of Ecology and Nature Conservation, Beijing Forestry University, Beijing 100083, China; CAS Key Laboratory of Forest Ecology and Silviculture, Institute of Applied Ecology, Chinese Academy of Sciences, Shenyang 110016, China; School of Ecology and Nature Conservation, Beijing Forestry University, Beijing 100083, China

**Keywords:** wood-inhabiting fungi, *Porodaedalea mongolica*, *Porodaedalea schrenkiana* secreted carbohydrate-active enzymes, secondary metabolites

## Abstract

*Porodaedalea* species, members of the order Hymenochaetales, are widely distributed in the temperate regions of the Northern Hemisphere and are known for their saprophytic, pathogenic, and medicinal properties. Currently, genomics has become a mainstream approach for exploring the potential applications of fungi, but the genomics studies on *Porodaedalea* remain extremely limited. In this study, we employed third-generation sequencing technology to obtain high-quality genomes of *Porodaedalea mongolica* and *Porodaedalea schrenkiana* using an assembly strategy optimized for assembling highly heterozygous genomes. Functional annotation and comparative genomic analyses revealed the lignocellulose decomposition strategies of *Porodaedalea* species, and explored their potential biological functions and patterns of their evolution history. These genomic resources provide the valuable foundation for future potential applications of the *Porodaedalea* species in the industrial and medical fields.

## Introduction

The genus of *Porodaedalea* Murrill belongs to Basidiomycota, Hymenochaetales, Hymenochaetaceae, which is widely distributed in the temperate regions of the Northern Hemisphere (Wu et al. 2019a; Yuan et al. 2023; Zhao et al. 2024a). It is mainly parasitic on the living trees, fallen trunks, and branches of conifers (Wu and Yuan 2020). These fungi act as decomposers in forest ecosystems, degrading lignocellulose in lodging trunks and branches by secreted carbohydrate-active enzymes (CAZymes), thus contributing to nutrient recycling and ecosystems sustainability (Worrall et al. 2005; Dai 2010a; Wu and Yuan 2020; Zhao et al. 2024b). *Porodaedalea* species are also significant forest pathogens, causing white rot of living conifers, resulting in economic losses (Dai 2010b; Szewczyk et al. 2017). For example, *Porodaedalea pini* (Brot.) Murrill can cause red ring rot with tree heartwood (Szewczyk 2015), infesting approx. 8% of Scots pine wood in Poland (Szewczyk 2008). In addition, some species have high medicinal value and are recognized as important medicinal fungi (Wu et al. 2019b; Ghobad-Nejhad et al. 2024). Some species can produce abundant secondary metabolites with potential pharmacological applications. For example, Yang et al. (2017) extracted 8 benzenoids from *P. pini*, including 3 new phenols [piniphenols A-C (1-3)]. Dogan et al. (2018) found that *P. pini* has important anti-herpes simplex virus 1 (HSV-1) activities by extracting secondary metabolites. Deveci et al. (2019) and Kaur et al. (2019) found that the secondary metabolites of *P. pini* had cytotoxic, antioxidant, and anticholinesterase activities. Sárközy et al. (2020) isolated 3 compounds with considerable antioxidant effect by chemical analysis of the methanol extract of *Porodaedalea chrysoloma* (Fr.) Fiasson and Niemelä.

With the continuous development of DNA sequencing technologies has greatly accelerated in molecular phylogeny and genomics research on wood-inhabiting fungi. The species number of *Porodaedalea* has been increased to 20 by using phylogenetic analyses and morphologic features (Dai et al. 2017; Wu et al. 2019a; Wu and Yuan 2020; Wu et al. 2022). The genomic data have been applied in the medical research related to fungal metabolites (Wu et al. 2015; Kiss et al. 2019; Chen et al. 2023; Jia et al. 2024). However, compared to other fungal groups, genomics research on *Porodaedalea* remains limited. Chung et al. (2017) conducted a comparative genomic analysis using the genome of *P. pini* alongside 3 other wood-pathogenic fungi, identifying many gene families involved in lignin-degrading, thereby highlighting its white-rot capabilities. Lee et al. (2019) used the mitochondrial genome of *P. pini* and compared it with other 5 Basidiomycetes species to explore the effect of horizontal gene transfer and recombination. Zhao et al. (2022) reported the complete mitochondrial genome of *Porodaedalea mongolica* Y.D. Wu and Y. Yuan, and Zhao et al. (2023a) assembled and annotated the genomes of *Porodaedale occidentiamericana* Y.C. Dai and F. Wu and *Porodaedalea qilianensis* Y.C. Dai and F. Wu, comparing them with 29 other Hymenochaetales species to gain insight into the ecological diversity. Zhao et al. (2024b) used the second-generation genomes of 10 *Porodaedalea* species to study the phylogeny and biogeography of Hymenochaetales.

To date, genome data of *P. chrysoloma* (Fr.) Fiasson and Niemelä and *Porodaedalea niemelaei* M. Fisch. are available in the JGI database (https://genome.jgi.doe.gov/portal/, accessed on May 18, 2025), and the genome data of *P. pini* are download in the NCBI database (https://www.ncbi.nlm.nih.gov/datasets/genome/, accessed on May 18, 2025). However, *P. chrysoloma* and *P. niemelaei* are correctly classified as *Phellinus* Quél. according to Index Fungorum database (http://www.indexfungorum.org/Names/Names.asp, accessed on May 18, 2025). Moreover, Zhao et al. (2024b) released raw sequencing data for 10 *Porodaedalea* species only, which are of insufficient quality for in-depth genomic analysis. Therefore, only *P. pini* in *Porodaedalea* has published high-quality genomic data.

To address the lack of comprehensive genomic studies on *Porodaedalea*, *P. mongolica* and *Porodaedalea schrenkiana* Y.C. Dai and F. Wu are sequenced using both PacBio and Illumina platforms. By improving the traditional assembly strategy, the sequencing data with high heterozygosity were assembled, and finally obtained a high-quality genome. Functional annotation of the genomes infers the lignocellulose degradation strategies employed by *Porodaedalea* species and to explore their potential biological functions in depth. Comparative genomic analyses further clarified the evolutionary trends within the genus. These findings provide a solid foundation for future research into the industrial and medical applications of *Porodaedalea* species.

## Materials and methods

### Strain acquisition, culture, and DNA extraction

The basidiomata of *P. mongolica* Dai 20809 were collected from the living tree of *Larix gmelinii* (Rupr.) Kuzen. in Hongwei Town, Huzhong District, Heilongjiang Province on August 27, 2019. *Porodaedalea schrenkiana* Dai 20232 were collected from the living tree of *Picea schrenkiana* Fisch. and C. A. Mey. in Tianshan Tianchi National Forest Park, Xinjiang Uygur Autonomous Region on August 23, 2019. Specimens were preserved in the herbarium of Beijing Forestry University (BJFC). The basidiomata were washed twice with 75% ethanol solution, and then rinsed 3 times with sterile water. The basidiomata with a size of about 5 mm^2^ were placed on potato dextrose agar (PDA) culture medium plates and cultured in darkness at 25 °C for 5 to 7 d. After the growth of mycelium near basidiomata, the 5 mm^2^ fungus block with mycelium at the edge of the colony was picked up and inoculated on new PDA culture medium plates. After 1 to 2 wk of dark culture at 25 °C, an axenic single colony was formed. The strain was preserved in BJFC at 4 °C. The surface hyphae were scraped off for genomic DNA extraction by the modified CTAB method (Liu et al. 2022). The purity and integrity of DNA were detected by agarose gel electrophoresis.

### Library construction and genome sequencing

The SMRTbell library was constructed using the SMRTbell TM Template kit 2.0 (Pacific Biosciences, Menlo Park, CA, United States). The DNA samples that were qualified by electrophoresis were disrupted with Covaris g-TUBE to obtain the target fragments of the size required for the construction of the library. After DNA damage repair and end repair, the hairpin adaptor was ligated to both ends of the DNA fragment using DNA adhesive enzyme, and the DNA fragment was purified using AMpure PB beads. The Blue Pippin fragment was used to screen for fragments of a specific size. The SMRTbell library was screened by AMpure PB beads, and then the DNA damage was repaired. The SMRTbell library was purified by AMpure PB beads again. The constructed library was quantified by Qubit 1× dsDNA HS Assay Kit (Thermo Fisher, Waltham, MA, United States) concentration, and the size of the inserted fragment was detected by Agilent 2100 (Agilent, Santa Clara, CA, United States) to complete the library construction of the PacBio platform.

The qualified DNA samples were randomly disrupted into fragments of about 350 bp by Covaris E200 ultrasonic disruptor (Covaris, Woburn, MA, United States). The DNA fragments after treatment were prepared using the NEBNext Ultra DNA Library Prep Kit for Illumina kit (New England Biolabs, Ipswich, MA, United States). The whole library was prepared by end repair, A tail addition, sequencing adapter addition, purification, and PCR amplification. After the library construction was completed, Qubit 1× dsDNA HS Assay Kit was used for preliminary quantification, and the library was diluted to 2 ng/μl. Then, Agilent 2100 was used to detect the insert fragments of the library. After the insert size was in line with expectations, Q-PCR method was used to accurately quantify the effective concentration of the library to ensure the quality of the library and complete the Illumina platform library construction.

After the library inspection was qualified, the PacBio and Illumina platform library were sequenced by PacBio Sequel II and Illumina NovaSeq PE150 according to the effective concentration and the target offline data volume. Filter the low-quality reads and bacterial contamination in the original offline data to obtain the final clean data. The library construction and sequencing were performed by Novogene Co., Ltd. (Beijing, China).

### Genome resource survey and assembly

After obtaining clean reads from both second- and third-generation sequencing, the second-generation data use FastQC v0.12.1 (Andrews 2010) for the overall evaluation. Then, quality filtering was performed with Fastp v0.23.4 (Chen et al. 2018), which included removing reads with a mass value of <20, adapter sequences, reads shorted than 50 bp, and reads containing N bases > 10. *k*-Mer analysis was performed using filtered second-generation data (K = 17). The *k*-mer frequency was counted using Jellyfish v2.3.0 (Marçais and Kingsford 2011), and the genome survey carried out using GenomeScope v2.0 (Vurture et al. 2017).

Third-generation sequencing data were assembled using Canu v2.2, Flye v2.9-b1778, and SMRT Link v5.0.1 with default parameters (Koren et al. 2017; Ardui et al. 2018; Kolmogorov et al. 2019). *k*-Mer analysis uncovered high heterozygosity in the sequencing data, and the initially estimated genome size appeared inaccurate. Therefore, the genome of *P. pini* BCRC 35384 was selected as a reference, and the genome reference size was set to 50 Mb. After each software completed these assemblies, the results were aligned to the third-generation sequencing data using minimap v2.24-r1122 (Li 2018), and the redundant sequences were removed with purge_dups v1.2.5 (Guan et al. 2020). The assembly outputs were then merged in multiple rounds using Quickmerge v0.3 (Chakraborty et al. 2016). To improve assembly accuracy, 4 rounds of polishing were performed on the final Quickmerge results using NextPolish v1.4 (Hu et al. 2020a) with both third-generation reads and Fastp-filtered second-generation reads. SeqKit v2.2.0 (Shen et al. 2016) was then used to manually remove contigs shorter than 150 Kb, yielding the final genome assembly. Genome quality and completeness were evaluated using QUAST v5.0.2 (Gurevich et al. 2013) and BUSCO v5.2.0 (database using https://busco-data.ezlab.org/v5/data/lineages/fungi_odb10.2024-01-08.tar.gz; Simão et al. 2015), respectively. In addition, the well-assembled genome of the closely related species *Pyrrhoderma noxium* (Corner) L.W. Zhou and Y.C. Dai (contigs of 13, N50 value of 2.74 Mb, and L50 value of 5) was used for comparative evaluation at each step.

### Genome prediction

For genome repeat sequence prediction, homology-based methods were performed using RepeatMasker v4.1.4 and RepeatProteinMask v4.1.4 (Tarailo-Graovac and Chen 2009) based on the RepBase database (https://www.girinst.org/repbase/) and Dfam database (https://www.dfam.org/home). Long terminal repeat (LTR) elements were predicted using LTRharvest (http://genometools.org/), LTR_FINDER v1.0.7 (Xu and Wang 2007), and LTR_retriever v2.9.0 (Ou and Jiang 2018). The prediction results were used to mask the LTR in the homology-based annotation results. For de novo prediction, RepeatModeler v2.0.3 (Flynn et al. 2020) was applied, followed by tandem repeat prediction using TRF v4.0.9 (Benson 1999). All annotation outputs were merged to generate the final repeat annotation set. For noncoding RNA (ncRNA), Infernal v1.1.4 (Nawrocki and Eddy 2013) was used with the Rfam database (https://rfam.org/). rRNA and tRNA were predicted using RNAmmer v1.2 (Lagesen et al. 2007) and tRNAscan-SE v2.0.9 (Chan et al. 2021), respectively. The rRNA and tRNA in the Infernal annotation results were shielded and combined with the prediction results to obtain the final ncRNA annotation results.

Repeat sequences in the genome were masked using Bedtools v2.30.0 (Quinlan 2014) to reduce their impact on gene structure annotation. Gene prediction was performed using a combination of 2 approaches, including *ab initio* with Augustus v3.3.3 (Stamatakis et al. 2008), homolog annotation with GeMoMa v1.9 (Keilwagen et al. 2016), and then integrated using EVM v1.1.1 (Haas et al. 2008). The agat_sp_filter_by_ORF_size.pl and agat_sp_filter_incomplete_gene_coding_models.pl in AGAT (https://agat.readthedocs.io/en/latest/) were used to filter out genes with open reading frames (ORFs) shorter than 50 amino acids and incomplete genes models, respectively. After renaming the gene name, the final annotation file was obtained. Coding sequences (CDS) files and protein sequence files were obtained using TransDecoder v5.5.0 (https://github.com/TransDecoder/TransDecoder) based on genome and annotation files. Signal peptides were predicted using SignalP v5.0 (Armenteros et al. 2019), and transmembrane domains were identified by TMHMM v2.0 (Moller et al. 2001) to obtained secretory proteins.

### Genome functional annotation

Protein-coding genes were annotated using Kyoto Encyclopedia of Genes and Genomes database (KEGG; Kanehisa et al. 2017), Non-Redundant Protein Sequence database (NR; https://www.ncbi.nlm.nih.gov/protein/), Eukaryotic Orthologous Group (KOG; Koonin et al. 2004), Fungal Cytochrome P450 database (FCPD; Moktali et al. 2012), Swiss-Prot database (Boeckmann et al. 2003), and Gene Ontology database (GO; Ashburner et al. 2000) databases with DIAMOND v2.0.15 (Buchfink et al. 2015), the cutoff values of E-value of ≤1e^−5^. Secreted CAZymes were identified using the online tool dbCAN3 (https://bcb.unl.edu/dbCAN2/blast.php; Zheng et al. 2023). Secondary metabolite gene clusters were predicted using antiSMASH v8.0 (https://fungismash.secondarymetabolites.org/; Blin et al. 2025) with the default parameters.

### Comparative genomics

Eight genomes of Hymenochaetales, 3 genomes of Polyporales, and *Auricularia cornea* were downloaded from the NCBI database (https://www.ncbi.nlm.nih.gov/). These species include white-rot and brown-rot fungi that grow on broad-leaved and coniferous trees, as well as they have been identified as medicinal or pathogenic fungi (Supplementary Table 1). Comparative analyses with these species were conducted for gene functional annotation, phylogenetic reconstruction, and gene family analysis. A heatmap of secreted CAZymes among species was constructed using Hiplot (https://hiplot.com.cn/). Gene families were clustered using OrthoFinder v2.5.4 (Emms and Kelly 2019) with default parameter to obtain single-copy orthologous (SCOs). These SCOs were aligned using ParaAT v2.0 (Zhang et al. 2012), cutting out nonconservative parts and merging them into supergene using SeqKit v2.2.0 and trimal v1.4.rev15 (Capella-Gutiérrez et al. 2009), respectively. A maximum likelihood phylogenetic tree was constructed using RAxML-NG v1.1.0 (Kozlov et al. 2019) under “GTR+G+I” model with 100 bootstraps replicates, and *A. cornea* was set as the outgroup. A time-calibrated phylogenetic tree was constructed using MCMCTREE in the PAML v4.10.0 (https://github.com/abacus-gene/paml/releases), employing the approximation likelihood method. Four calibration points were used following Zhao et al. (2024b): (i) *Auricularia* at 221 million years ago (Mya), (ii) the divergence between Hymenochaetales and Polyporales at 185 Mya, (iii) *Pyrrhoderma* at 113 Mya, and (iv) the split between *Inonotus* and *Sanghuangporus* at 61 Mya. After a burn-in of 2000 iterations, samples were collected every 100 iterations for a total of 200,000 iterations. Gene family contraction and expansion were performed using CAFE v.5.0 based on a clock-calibrated tree, specifying the root frequency distribution as Poisson distribution. The model parameter “-k” was tested across values from 2 to 5, and the result with the maximum final likelihood under the gamma model was selected as optimal. The trees were viewed using FigTree v1.4.4 (http://tree.bio.ed.ac.uk/software/figtree).

## Results

### Genome survey and component of *P. mongolica* and *P. schrenkiana*

The strains of *P. mongolica* Dai 20809 and *P. schrenkiana* Dai 20232 were sequenced using both the PacBio Sequel II and Illumina PE150 platform, yielding 20.99 and 13.47 Gb of raw data, respectively. According to the quality control reports from FastQC v0.12.1, the mean quality value of each base position in the reads of *P. mongolica* and *P. schrenkiana* ranged from 33.81 to 36.44 (Supplementary Fig. 1a and b). The GC contents of *P. mongolica* and *P. schrenkiana* were 50% and 51%, respectively. The proportion of repetitive sequences in the forward/reverse sequencing data were 16.9/17.7% for *P. mongolica* and 28.7/30.1% for *P. schrenkiana* (Supplementary Fig. 1c and d), respectively. The *k*-mer analysis showed that the maximum coverage depths of *P. mongolica* and *P. schrenkiana* were 43.4× and 36×, respectively, and the estimated genome sizes were 41.6 and 51.1 Mb, and the heterozygosity rates were 1.91% and 0.98%, respectively (Supplementary Fig. 2a and b).

**Fig. 1.**
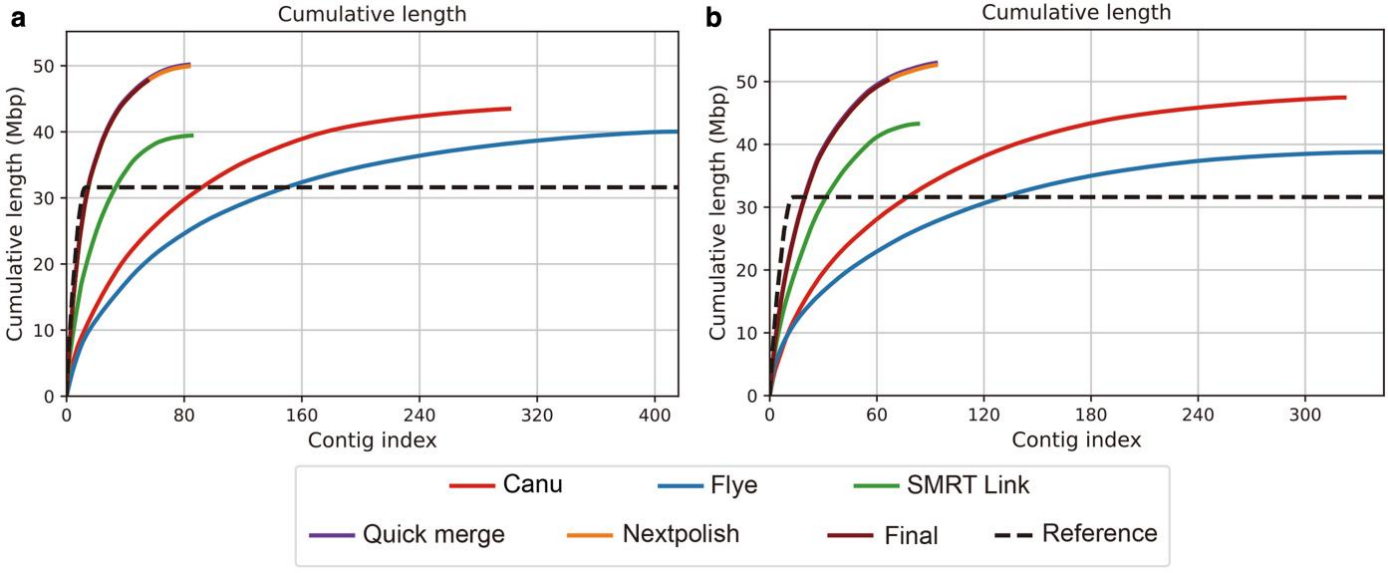
Continuity curves of genomes assemblies using different software. a) *Porodaedalea mongolica*; b) *P. schrenkiana*. The abscissa represents the number of contigs, and the ordinate represents the cumulative length. Contigs are ordered from largest to smallest. Final represents the final assembly result, Reference represents the *Pyrrhoderma noxium* genome.

Comparing the assembly results of each software, the best quality is selected for merged assembly, and the final assembly result with assembly contiguity close to *P. noxium* is obtained (Fig. 1). *Porodaedalea mongolica* was assembled into a total size of 46.26 Mb, consisting of 55 contigs, with a GC content of 50.02%, N50 value of 1.9 Mb, L50 value of 9, and largest contigs of 4.14 Mb. The genome completeness was evaluated at 96% using BUSCO v5.2.0 with fungi_odb10 database (Table 1). *P. schrenkiana* was assembled into a total size of 48.65 Mb, consisting of 66 contigs, with GC content of 50.25%, N50 value of 1.2 Mb, L50 value of 16, largest contigs of 3.11 Mb, and the genome completeness score was 96.1% assessed by BUSCO v5.2.0 fungi_odb10 database (Table 1).

**Table 1.** Statistics of the genome assembly and component of *Porodaedalea mongolica* and *P. schrenkiana*.

Species	*P. mongolica* Dai 20809	*P. schrenkiana* Dai 20232
Genome size (Mb)	46.26	48.65
Contigs	55	66
Largest contigs (Mb)	4.14	3.10
GC (%)	50.02	50.25
N50 (Mb)	1.90	1.20
L50	9	16
BUSCO completeness (%)	96.00	96.10
Repetitive elements (%)	23.7	34.16
ncRNA	251	303
Protein-coding genes	13,437	12,530

Using a combination of homology-based annotation and de novo prediction, the total length of repeat sequences in *P. mongolica* and *P. schrenkiana* was 10.92 and 17.39 Mb, accounting for 23.7% and 34.16% of the genome size, respectively (Table 1). The main repeat element was LTRs, accounting for 13.37% and 20.98% of the genomes, respectively. By integrating results from multiple ncRNA prediction software, the *P. mongolica* genome contained 22 snRNAs, 6 rRNAs, and 196 tRNAs. The *P. schrenkiana* genome consisted of 24 snRNAs, 7 rRNAs, and 262 tRNAs (Table 1). *Porodaedalea mongolica* and *P. schrenkiana* predicted 13,437 and 12,530 protein-coding genes, respectively, based on *ab initio* prediction and homolog annotation (Table 1), with an average CDS length of 1,434 and 1,420 bp. The number of annotated protein genes of *P. mongolica* and *P. schrenkiana* in NR database was the highest (10,723 vs 9,973), followed by GO (10,425 vs 9,540), SwissProt (6,707 vs 6,201), KOG (4,533 vs 4,136), KEGG (3,168 vs 2,904), FCPD (130 vs 137), and secreted CAZymes (185 vs 186).

### Degradation strategy of lignocellulose by *Porodaedalea*

In *P. mongolica*, *P. pini*, and *P. schrenkiana*, a total of 185, 169, and 186 genes, respectively, were predicted to encode secreted CAZyme families (Table 2). Among these species, *P. pini* exhibited the lowest total number of secreted CAZymes genes, primarily due to the absence of glycoside hydrolase (GH) family genes (Table 2). Additionally, *P. schrenkiana* possessed more auxiliary activity (AA) family genes than *P. pini* and *P. mongolica* (Table 2). By comparing the total number of secreted CAZymes across all analyzed species, the total number of secreted CAZymes genes of *Porodaedalea* species was less than that of white-rot fungi hosted by broad-leaved branches in Polyporales and Hymenochaetales, the main difference contributing to this disparity was the reduced number of AA family genes in the *Porodaedalea* species (Table 2).

**Table 2.** Statistics of secretory CAZymes annotation results of 13 woody fungi.

Species	AA	CBM	CE	GH	GT	PL	Total
*Daedaleopsis sinensis*	79	23	19	126	4	9	260
*Ganoderma sinense*	74	34	20	196	7	8	339
*Rhodofomes roseus*	19	8	7	119	4	3	160
*Fomitiporia mediterranea*	58	16	13	114	4	6	211
*Fomitiporia polymorpha*	63	16	12	118	5	8	222
*Inonotus obliquus*	43	17	10	105	4	8	187
*Porodaedalea mongolica*	32	16	11	117	1	8	185
*Porodaedalea pini*	36	17	8	100	1	7	169
*Porodaedalea schrenkiana*	43	19	7	110	1	6	186
*Pyrrhoderma noxium*	44	16	14	81	2	10	167
*Sanghuangporus baumii*	45	14	11	104	2	8	184
*Sanghuangporus sanghuang*	54	15	11	114	4	8	206
*Sanghuangporus weigelae*	45	18	11	116	3	7	200

AA, auxiliary activities; CBM, carbohydrate-binding modules; CE, carbohydrate esterases; GH, glycoside hydrolases; GT, glycosyltransferases; PL, polysaccharide lyases.

**Fig. 2.**
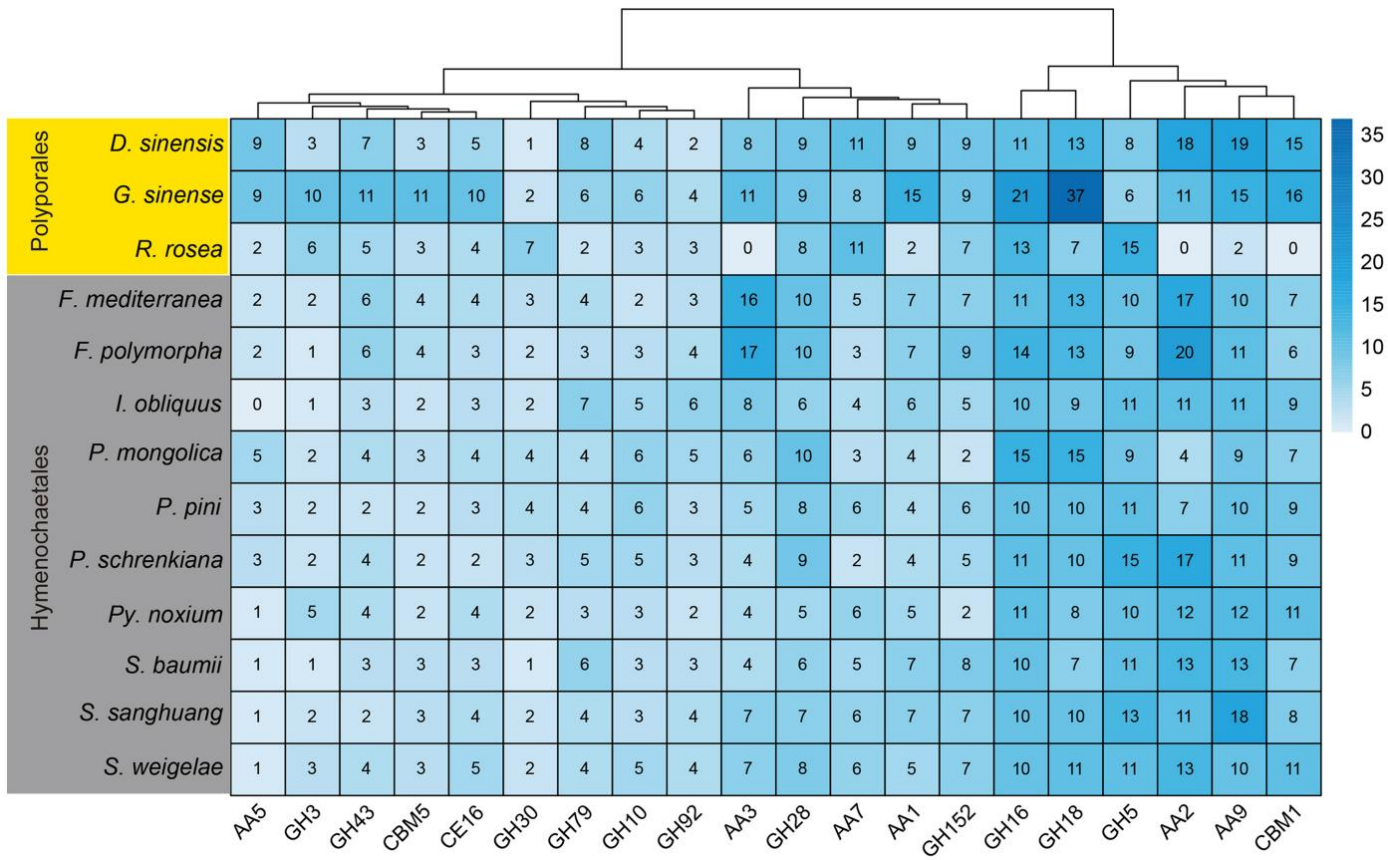
The secreted CAZymes with a number more than 5 in 13 woody fungi. AA, auxiliary activities; CBM, carbohydrate-binding modules; CE, carbohydrate esterases; GH, glycoside hydrolases.

Among the secreted CAZymes with more than 5 numbers in *Porodaedalea*, *P. mongolica* had a greater number of GH16 and GH18 family genes (Fig. 2). The members of the GH16 family genes are widely distributed across diverse taxonomic groups and primarily function in the degradation or remodeling of cell wall polysaccharides. They mainly target hemicellulose components such as *β*-1,3-glucan, *β*-1,4-glucan, xyloglucan, and xylan. The GH18 family genes mainly hydrolyzes chitin components such as *β*-1,4 glycosidic (Sørbotten et al. 2005), and chitin is the main component of plant cell wall (Gong et al. 2020). Although GH18 family genes can’t directly encode enzymes capable of degrading lignocellulose, they can accelerate the efficiency of lignin peroxidase (LiP), manganese peroxidase (MnP), laccase to decompose lignin by destroying plant cell wall (Duan et al. 2003). *Porodaedalea schrenkiana* had more AA2 and GH5 family genes (Fig. 2). AA2 family genes contain class II lignin-degrading peroxidases that catalyzed many oxidation reactions using H_2_O_2_ or organic peroxides as electron acceptors (Ma et al. 2024a, 2024b). GH5 family genes, known as “cellulase family A”, were one of the largest glycoside hydrolase families. They had a wide range of specificities and is extremely rich in various ecological niches (Aspeborg et al. 2012). However, *P. pini* does not have prominent secreted CAZymes (Fig. 2), which may be associated with its ability to simultaneously degrade all major components of wood (Sunardi et al. 2016) (Table 2).

**Fig. 3.**
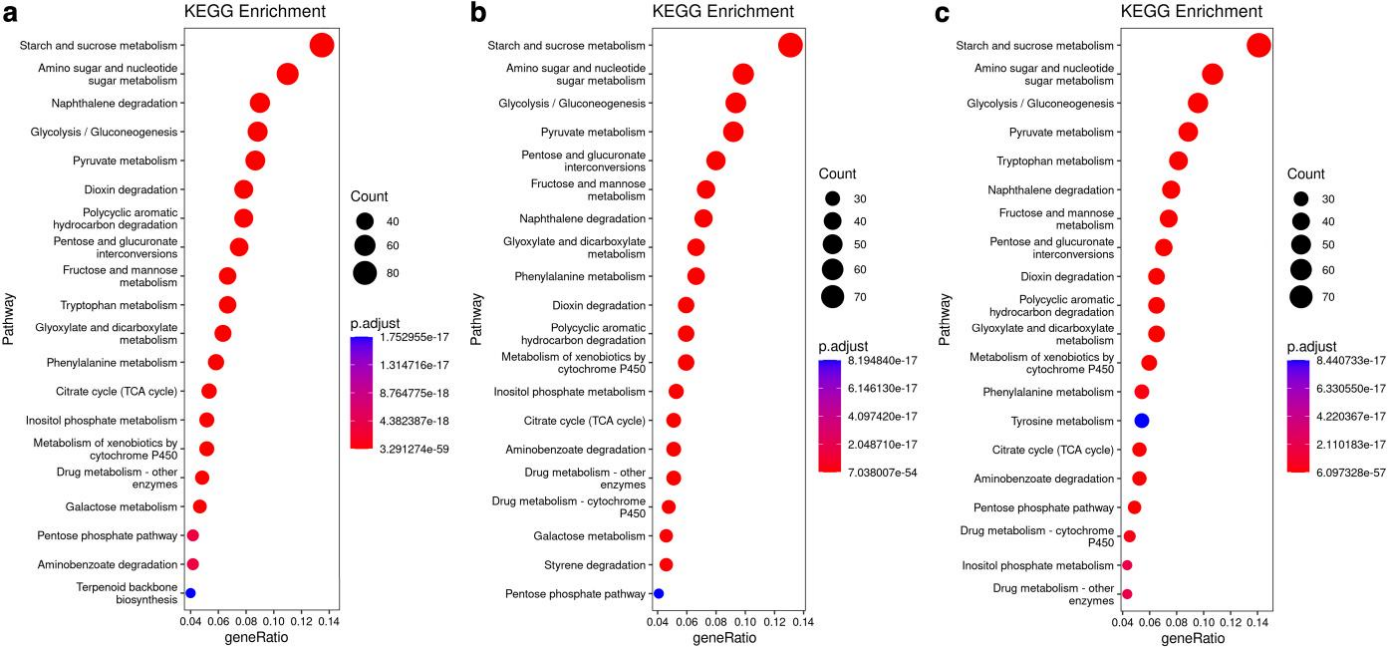
KEGG pathway enrichment bubble plots. a) *Porodaedalea mongolica*; b) *P. schrenkiana*; c) *Porodaedalea pini.* Bubble color represents the degree of enrichment. Bubble size corresponds to the number of genes associated with each pathway.

In addition, KEGG pathway enrichment analysis was performed based on the annotated genes of *Porodaedalea* species, in which the starch and sucrose metabolism pathway related to cellulose and hemicellulose degradation was the most significantly enriched pathway involved in the genes (Fig. 3). A total of 81, 78, and 77 genes were involved in this pathway in *P. mongolica*, *P. schrenkiana*, and *P. pini*, respectively. Among them, 13, 11, and 11 genes encode *β*-glucosidase, as well as 6, 7, and 5 genes encode 1,4-*β*-cellobiosidase, respectively. 1,4-*β*-cellobiosidase catalyzed the hydrolysis of *β*-1,4-glycosidic bonds to release cellobiose, while *β*-glucosidase converted cellobiose into glucose. The main components of hemicellulose are xylan and mannan (Dhawan and Kaur 2007). In *Porodaedalea* species, galactose metabolism, fructose and mannose metabolism, pentose, and glucuronate interconversions pathways were significantly enriched (Fig. 3), suggesting their potential roles in hemicellulose glycan degradation. Lignin is a highly heterogeneous and resistant aromatic polymer, accounting for 10% to 30% of the total lignocellulosic biomass (Wang et al. 2015). Polycyclic pathways, such as aromatic hydrocarbon degradation, phenylalanine metabolism, pyruvate metabolism, and aminobenzoate degradation, were significantly enriched in *Porodaedalea* species, indicating their possible involvement in the direct or indirect breakdown of lignin (Fig. 3).

### Preliminary evidence of environmental pollutant degradation capability in *Porodaedalea*

Cytochrome P450 (CYPs) are known to facilitate the degradation of environmental pollutants and the synthesis of bioactive metabolites through various biochemical reactions, such as hydroxylation, epoxidation, dealkylation, desulfidation, dehalogenation, and nitroreduction (Silva et al. 2003; Chigu et al. 2010; Cook et al. 2016; Zeng et al. 2016; Ma et al. 2024a). In the FCPD annotation results, CYPs in *Porodaedalea* species were classified into 6 categories. Among them, 82, 70, and 67 protein-coding genes in *P. mongolica*, *P. schrenkiana*, and *P. pini*, respectively, were annotated as “E-class P450, group I”—the largest CYP class in these fungi. CYP51, CYP61, and CYP63 are common gene families in white-rot fungi (Dacco et al. 2020).

In the KEGG annotation results of *Porodaedalea* species, several pathways significantly enriched were associated with the degradation of environmental pollutants (Fig. 3). In the pathways of polycyclic aromatic hydrocarbon degradation, naphthalene degradation, and dioxin degradation, *P. mongolica*, *P. pini*, and *P. schrenkiana* have 47, 36, and 35 genes, respectively, encoding salicylate hydroxylase. Salicylic acid is an important raw material in the production of pharmaceuticals, food additives, and rubber materials, has become a pervasive organic pollutant in aquatic and terrestrial environments due to its widespread industrial application (Karunanayake et al. 2017; Ma et al. 2024b). Salicylate hydroxylase is a key oxidase for the degradation of aromatic pollutants, catalyzing salicylic acid to introduce hydroxyl groups at the ortho position of the benzene ring to form catechol, which can be further oxidized by laccase or peroxidase to produce *β*-ketoadipic acid through the ortho cleavage pathway. Finally, it enters the tricarboxylic acid (TCA) cycle and mineralizes into CO_2_ and H_2_O (Ma et al. 2024b). In addition, pathways such as styrene degradation, aminobenzoate degradation and metabolism of xenobiotics by cytochrome P450 that have the ability to degrade environmental pollutants also showed significant enrichment (Fig. 3).

### *Porodaedalea* can produce a variety of secondary metabolites

In fungal genome research, gene annotation through KEGG pathways provides valuable insights into the biological functions and molecular interactions underlying fungal metabolism. Among the significantly enriched KEGG pathways in *Porodaedalea* species, the starch and sucrose metabolism pathway was predominantly associated with the biosynthesis of fungal polysaccharides (Fig. 3). *Porodaedalea mongolica*, *P. pini*, and *P. schrenkiana* had 12, 15, and 12 genes, respectively, encoding 1,3-*β*-glucosidase. 1,3-*β*-glucans were not only the main components of fungal cell wall, but also have immunomodulatory and antioxidant functions (Shao et al. 2020; Zhao et al. 2023b). In addition, the terpenoid backbone biosynthesis pathway was slightly enriched in *P. mongolica* (Fig. 3), whereas no significant enrichment was observed in *P. pini* and *P. schrenkiana*, indicating that *P. mongolica* had stronger terpenoid synthesis ability than *P. pini* and *P. schrenkiana*.

The secondary metabolic biosynthetic gene cluster (BCGs) of *Porodaedalea* were compared with those of medicinal fungi *Ganoderma sinense* J.D. Zhao et al., *Sanghuangporus baumii* (Pilát) L.W. Zhou and Y.C. Dai, *Sanghuangporus sanghuang* (Sheng H. Wu, et al.) Sheng H. Wu, et al., and *Sanghuangporus weigelae* (T. Hatt. and Sheng H. Wu) Sheng H. Wu, et al. The results revealed that *Porodaedalea* species had a greater number of terpene BCGs—responsible for terpenoid synthesis—than *Sanghuangporus* species (Table 3). Notably *P. mongolica* and *P. schrenkiana* had higher terpene BCGs than *G. sinense*. In addition, the number of non-ribosomal peptide synthetase-like (NRPS-like) gene clusters in *Porodaedalea* species was higher than in *Sanghuangporus* species (Table 3), indicating that *Porodaedalea* species also had the ability to synthesize aromatic polyketides.

**Table 3.** The annotation results of secondary metabolic biosynthetic gene clusters of *Porodaedalea* species and 3 medicinal fungi.

Species	Terpene	NRPS-like	T1PKS	Others	Total
*Ganoderma sinense*	25	8	3	5	41
*Porodaedalea mongolica*	28	8	6	3	45
*Porodaedalea pini*	24	6	4	3	37
*Porodaedalea schrenkiana*	29	6	4	6	45
*Sanghuangporus baumii*	20	3	3	3	29
*Sanghuangporus sanghuang*	26	4	3	4	37
*Sanghuangporus weigelae*	19	4	3	3	29

Terpene, terpenoid biosynthetic; NRPS-like, non-ribosomal peptide synthetase-like; T1PKS, type I polyketide synthase.

### Phylogenetic and gene family analysis of *Porodaedalea*

**Fig. 4.**
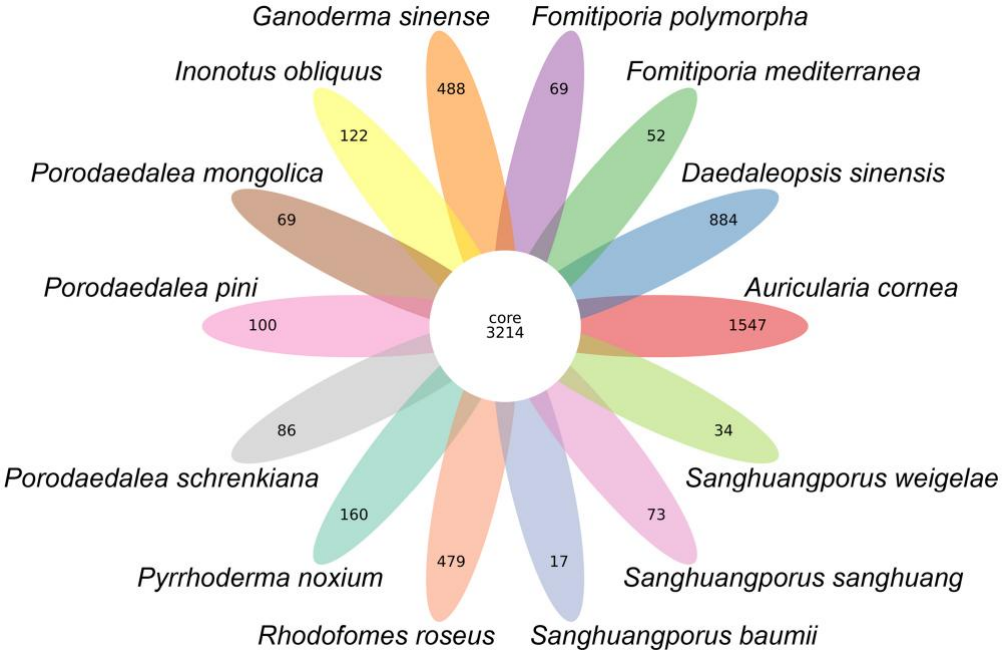
The gene family petals of 14 species, the center of the petals represents the number of common genes.

**Fig. 5.**
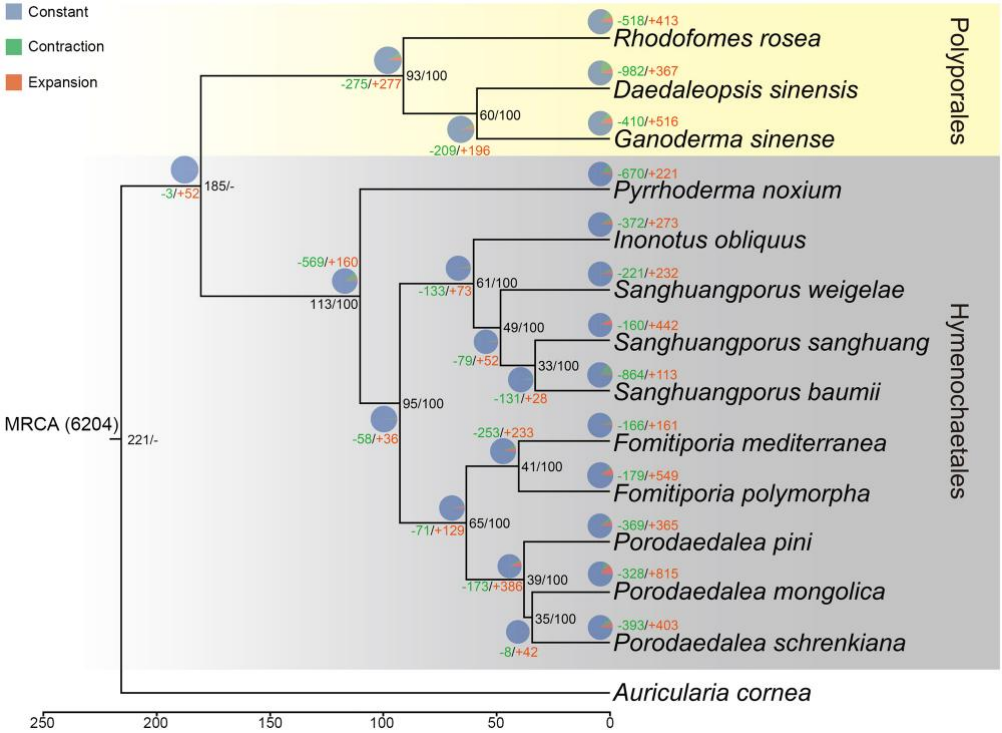
Time-calibrated phylogenetic tree was constructed using a single-copy gene orthologous sequence with *Auricularia cornea* as the outgroup. The branches represent divergence times in million years. The left side of the font of the branch node is the divergence time, and the right side is bootstrap support. The branch node and the color fonts above the species are the number of gene families of contraction and expansion, respectively.

A total of 3,214 core gene families were identified as shared among 14 species in this study (Fig. 4). *Porodaedalea mongolica*, *P. pini*, and *P. schrenkiana* had 69, 100, and 86 unique gene families, respectively (Fig. 4). In order to further understand the species evolution process of *Porodaedalea* species, a maximum likelihood (ML) tree was constructed using 1,214 single-copy orthologous genes. It was suggested that *Porodaedalea* was closest relative to *Fomitiporia*, with diverged around 65 Mya (Fig. 5). In *Porodaedalea*, *P. pini* diverged around 39 Mya, *P. mongolica* and *P. schrenkiana* diverged around 35 Mya (Fig. 5), which is consistent with the finding of Zhao et al (2024b).

**Fig. 6.**
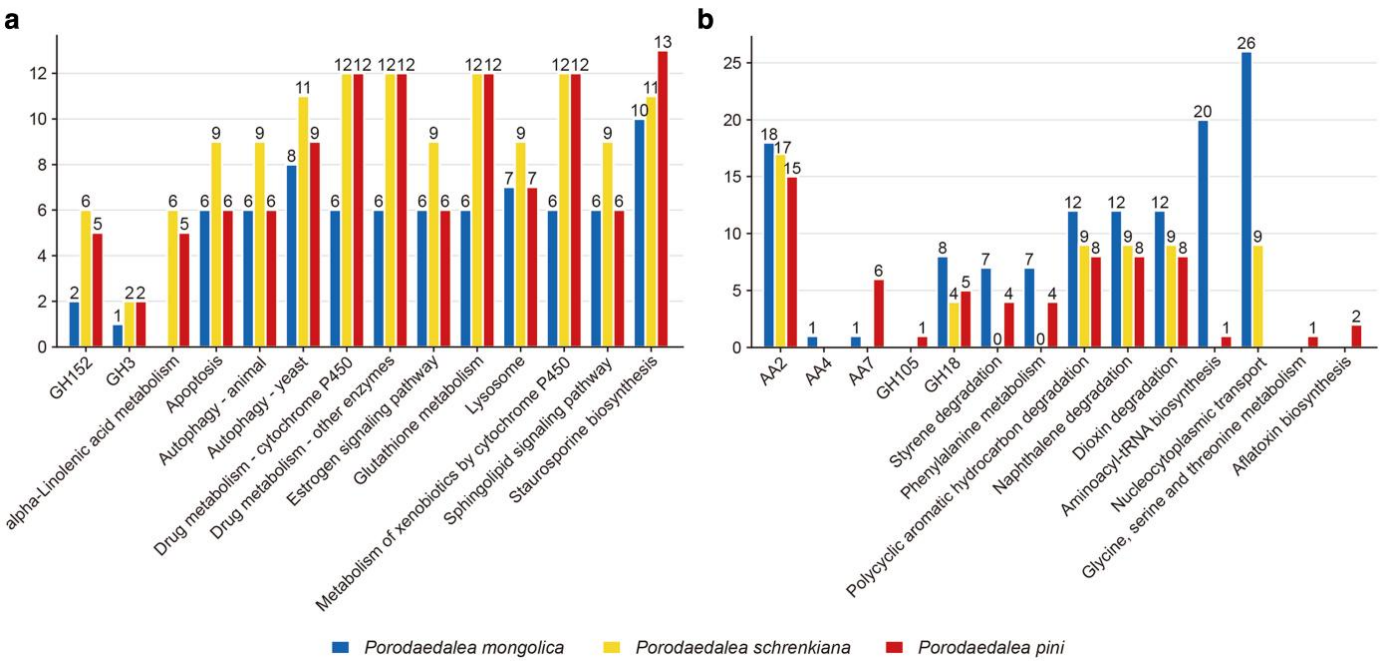
*Porodaedalea* species contraction and expansion gene family corresponding gene CAZymes and KEGG annotation results histogram. a) contracted of CAZymes gene families and KEGG pathways; b) expanded CAZymes gene families and KEGG pathways. AA, auxiliary activities; GH, glycoside hydrolases.

In this study, a total of 6,204 gene families of 15 species were analyzed for contraction and expansion. 328, 369, and 393 gene families of *P. mongolica*, *P. pini*, and *P. schrenkiana* were contracted, while 815, 365, and 403 gene families were expanded, respectively (Fig. 5). The genes associated with contracted and expanded gene families in *Porodaedalea* species were annotated for CAZyme classes and KEGG pathways. It was found that GH152 and GH3 families in *Porodaedalea* species were contracted, while the AA2 family significantly was expanded (Fig. 6). This suggests that *Porodaedalea* species have continuously enhanced the lignocellulose decomposition capacity through increased peroxidase secretion. In KEGG enrichment analysis, the significantly contracted pathways included drug metabolism-cytochrome P450, drug metabolism-other enzymes, glutathione metabolism, metabolism of xenobiotics by cytochrome P450 (Fig. 6). In contrast, expansion was observed in pathways mainly included polycyclic aromatic hydrocarbon degradation, naphthalene degradation, and dioxin degradation (Fig. 6); these pathways are related to salicylate hydroxylase. Moreover, only *P. mongolica* was significantly expanded in aminoacyl-tRNA biosynthesis and nucleocytoplasmic transport pathways.

## Discussion

In the life cycle of Basidiomycota fungi, most species that are dominated have a dikaryotic stage with clamp connection, while a few species maintain a dikaryotic stage without clamp connection, and rare species are monokaryotic. Dikaryon represents a typical heterokaryotic, characterized by mycelial cells containing 2 compatible haploid nuclei (Bao 2024; Bao et al. 2024). In fungal genomics, obtaining monokaryotic hyphae through single-spore isolation is considered ideal for genome sequencing, as it significantly reduces genome heterozygosity and improves assembly quality. However, in practice, acquiring monokaryotic cultures is considerably more challenging than obtaining dikaryotic ones. In this study, the sequencing data showed a high heterozygosity rate. To address this problem, we used purge_dups v1.2.5 to remove the redundancy sequences of the preliminary assembly results. Although this reduced the number of contigs, it also resulted in decreased completeness. To compensate, we employed Quickmerge v0.3 to make up for this shortcoming by using high-completeness assembly results as a supplement. In addition, the results found that deletion of sequences shorter than 100 Kb did not affect gene completeness in genomes with high heterozygosity. By constantly adjusting the deletion length, gene completeness is maintained within an acceptable range, which can reduce the number of contigs and increase the N50 value.

The degradation patterns of wood components vary among different species of white-rot fungi and wood types (Eriksson et al. 1990; Tanaka et al. 2009). The results of secreted CAZymes revealed that the 3 *Porodaedalea* species adopted distinct strategies for lignocellulose degradation. *Porodaedalea mongolica* mainly infects *Larix* spp., *P. schrenkiana* is associated with *Picea* spp., and *P. pini* is distributed across various coniferous trees (Szewczyk 2015; Wu and Yuan 2020; Wu et al. 2022). The heartwood of *Larix* spp. is rich in terpene resins (such as pinoresinic acid), and the high condensation structure of lignin forms a stable barrier in the later stage of decomposition, while the size of peroxidases is too large to penetrate into the pores of wood cell wall (Messner and Srebotnik 1994; Ostroukhova et al. 2012). In response, *P. mongolica* has a large number of GH18 family genes, which can disrupt the cell wall structure and enlarge the pore size. This may promote the diffusion of peroxidase secreted by wood of *P. mongolica* in *Larix* spp., which is conducive to the degradation of lignin. Moreover, coniferous trees typically contain higher and more variable levels of lignin and cellulose compared to broadleaf trees Hu et al. (2009). While guaiacyl lignin predominates in most conifers, *Picea* spp. also contain syringyl lignin, which requires oxidative cleavage of methoxy groups and aromatic ring opening for degradation (Xue and Nan 2016). There are more AA2 families in *P. schrenkiana*, which secreted more peroxidase. *Porodaedalea pini* thrives on a variety of conifer hosts, suggesting a generalist lignocellulose degradation strategy. This ecological flexibility may reduce the evolutionary pressure to specialize in secreting a dominant CAZyme family, allowing *P. pini* to degrade all major wood components simultaneously without a distinct secreted CAZyme (Sunardi et al. 2016).

*Porodaedalea pini* mainly relies on manganese(II)-dependent peroxidase to decompose lignin, and MnP is mainly encoded by AA2 family (Sunardi et al. 2016; Melo-Nascimento et al. 2018). In this study, the AA2 family of *Porodaedalea* species showed significant expansion, indicating that *Porodaedalea* have evolutionarily enhanced their ability to secrete peroxidases. Previous studies have suggested that factors such as environmental temperature, humidity, and soil pH directly affect fungal growth, enzyme activity, and pollutant degradation (Anton-Herrero et al. 2023; Vaksmaa et al. 2023). From this point, it is plausible that *Porodaedalea* species have migrated from harsh environments to more stable habitats, thereby reducing the evolutionary pressure to maintain an energy-intensive exogenous detoxification system. This may explain the observed contraction in detoxification-related pathways. In the significantly expanded pathways, many genes are involved in encoding salicylate hydroxylase. When considered alongside the contraction of exogenous detoxification pathways, this suggested that salicylate hydroxylase may not primarily function in degrading salicylic acid from the environment in *Porodaedalea*. However, functional studies on salicylate hydroxylase in fungi remain limited (Rabe et al. 2013; Ambrose et al. 2015; He et al. 2023).

*Porodaedalea* species can produce bioactive metabolites with demonstrated anti-inflammatory, antioxidant, and anticancer properties. They are used as the medicinal fungi in many Asian countries for the treatment of ailments such as gastrointestinal diseases, cardiovascular diseases, and diabetes (Devi et al. 2022). Current studies on medicinal active ingredients in *Porodaedalea* species basidiomata primarily employ methanol or water extraction, focusing on natural phenolic substances (Deveci et al. 2019; Kaur et al. 2019; Devi et al. 2022). Terpenoids and polysaccharides were 2 major pharmacologically active compounds found in medicinal fungi (Cai et al. 2019; Sułkowska-Ziaja et al. 2023), For example, *Ganoderma lucidum* (Curtis) P. Karst. contains ganoderma-triterpenoids produced with immunomodulatory, antitumor, and liver-protecting functions, while *Macrolepiota procera* (Scop.) Singer polysaccharide components with anticancer effects (Seçme et al. 2018; Zeng et al. 2018; Hu et al. 2020b; Ryu et al. 2021). In this study, functional annotation of *Porodaedalea* species showed that a relatively high number of terpene gene clusters involved in terpenoid biosynthesis, and pathways related to the biosynthesis of polysaccharides were also significantly enriched. These findings suggest that Porodaedalea species harbor a wide array of yet unidentified terpenoid and polysaccharide metabolites, which may contribute to their medicinal properties and warrant further biochemical and pharmacological investigation.

## Conclusion

This study provides high-quality genomes of *P. mongolica* and *P. schrenkiana* for the first time, expanding the genome resources of *Porodaedalea*, and providing an assembly strategy for assembling highly heterozygous genomes. Through in-depth genomics analysis of *Porodaedalea*, the strategy of *Porodaedalea* to decompose lignocellulose was explored. Functional annotation of *Porodaedalea* genomes revealed that their potential to degrade environmental pollutants and produce secondary metabolites, such as terpenoids and polysaccharides. Phylogenetic reconstruction combined with gene family analysis clarified the divergence time of *Porodaedalea* and highlighted patterns of gene family contraction and expansion throughout its evolutionary history, offering important insights into the lineage's evolutionary dynamics. In general, *Porodaedalea* species can evolve different survival strategies for different parasitic environments, and have high application potential in future industrial and medical applications.

## Supplementary Material

jkaf195_Supplementary_Data

## Data Availability

The sequencing data and genome assembly project had been deposited in the NCBI database under the BioProject accession PRJNA1232978. Genome annotation files can be available at figshare: https://doi.org/10.6084/m9.figshare.29906249. Supplemental material available at G3 online.
