# Supplementary material for: High-quality genome assembly and annotation of Porodaedalea mongolica and Porodaedalea schrenkiana provide insights into potential industrial and medical application
Source: G3 (Bethesda). 2025 Sep 25;15(11):jkaf195. doi: 10.1093/g3journal/jkaf195 (PMC12611241; doi:10.1093/g3journal/jkaf195)
Supplement: jkaf195_Supplementary_Data [file jkaf195_supplementary_data.doc]

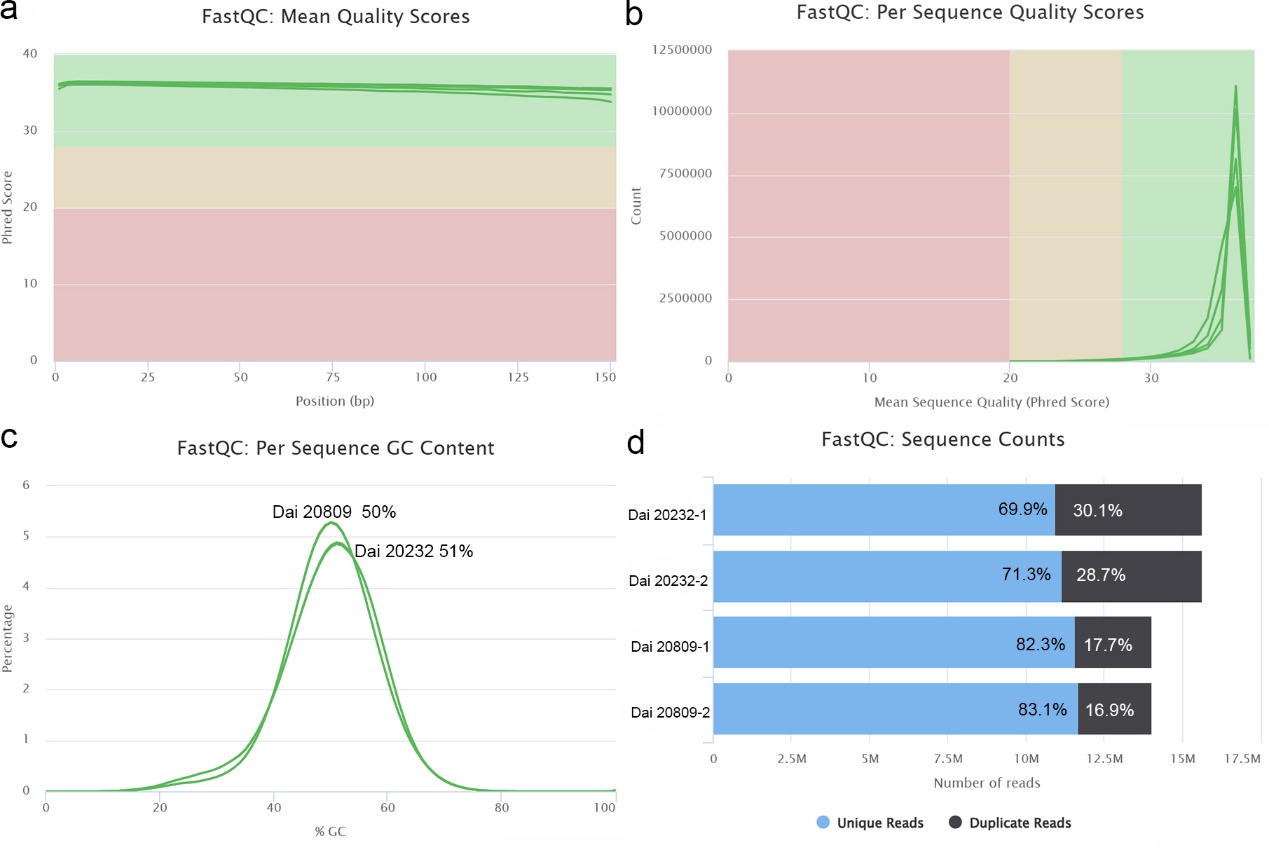


Fig. S1. Bioinformatics analysis of *Porodaedalea mongolica* and *P. schrenkiana* by FASTQC (a) Sequence quality histograms. The mean quality value across each base position in the read. (b) Per sequence quality scores. The number of reads with average quality scores. (c) Per Sequence GC Content. Normal random library typically have a roughly normal distribution of GC content. (d) Sequence counts. Sequence counts for each sample. Duplicate read counts are an estimate only.


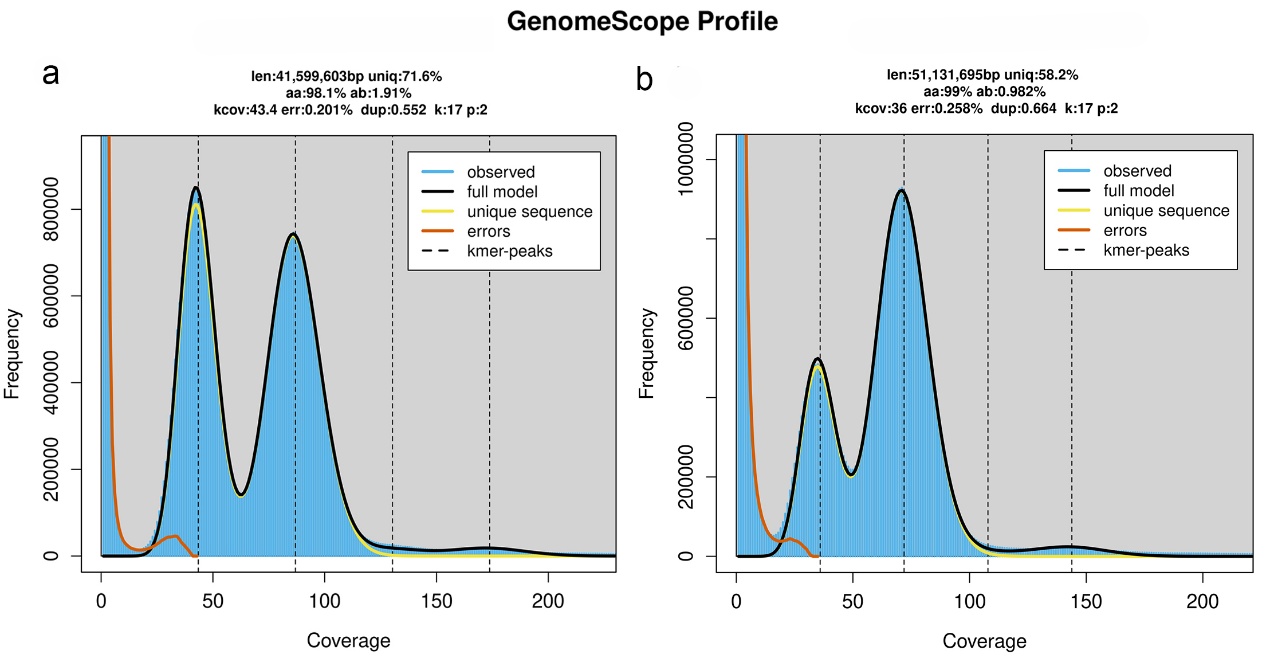


Fig. S2. Histogram of sequencing depth distribution (a) *Porodaedalea mongolica*; (b) *Porodaedalea schrenkiana*.

Table S1. Taxonomic Affiliation, Genomic Features of 12 Genomes Used in This Study

| Species | Sample no. | Host | Type of rot | Genome size (Mb) | Contigs | N50 (Mb) | L50 | BUSCOs in assembly (%) | GC (%) | Protein-coding genes | Literature cited |
| --- | --- | --- | --- | --- | --- | --- | --- | --- | --- | --- | --- |
| *Auricularia cornea* | ACW001 | broad-leaved | white-rot | 78.70 | 13 | 6.00 | 7 | 93.70% | 59.50 | 21,707 | Ma et al. 2023 |
| *Fomitiporia mediterranea* | CO36 | broad-leaved | white-rot | 62.25 | 58 | 5.41 | 4 | 95.50% | 40.35 | 10,687 | Garcia et al. 2024 |
| *Fomitiporia polymorpha* | WFB1 | broad-leaved | white-rot | 82.65 | 32 | 5.40 | 6 | 95.00% | 38.63 | 11,173 | Garcia et al. 2024 |
| *Pyrrhoderma noxium* | FFPRI411160 | broad-leaved & coniferous | white-rot | 30.52 | 13 | 2.74 | 5 | 95.00% | 41.57 | 9,959 | Chung et al. 2017 |
| *Sanghuangporus baumii* | 821 | broad-leaved | white-rot | 30.57 | 217 | 0.27 | 34 | 93.80% | 47.70 | 8,455 | Wang et al. 2022 |
| *Inonotus obliquus* | CFCC83414 | broad-leaved | white-rot | 35.03 | 32 | 3.10 | 5 | 96.20% | 47.48 | 10,450 | Hao et al. 2023 |
| *Porodaedalea pini* | BCRC 35384 | coniferous | white-rot | 51.47 | 220 | 0.57 | 25 | 95.50% | 49.27 | 12,869 | Chung et al. 2017 |
| *Sanghuangporus sanghuang* | VVIT01 | broad-leaved | white-rot | 34.50 | 37 | 2.51 | 6 | 95.00% | 47.96 | 10,597 | Wei et al. 2023 |
| *Sanghuangporus weigelae* | Si20 | broad-leaved | white-rot | 32.88 | 13 | 2.77 | 5 | 95.10% | 47.93 | 10,335 | Jin et al. 2024 |
| *Daedaleopsis sinensis* | Si85 | broad-leaved | white-rot | 50.10 | 24 | 4.40 | 6 | 96.10% | 56.48 | 18,828 | Ma et al. 2024 |
| *Ganoderma sinense* | ZZ0214-1 | broad-leaved | white-rot | 47.47 | 69 | 2.26 | 8 | 95.90% | 56.17 | 15,478 | Zhu et al. 2015 |
| *Rhodofomes roseus* | CIRM-BRFM 1785 | broad-leaved | brown-rot | 37.06 | 80 | 1.14 | 11 | 97.30% | 55.79 | 13,139 | Hage et al. 2021 |

**Literature cited**

Chung CL, Lee TJ, Akiba M, Lee HH, Kuo TH, Liu D, Ke HM, Yokoi T, Roa MB, Lu MYJ, et al. 2017. Comparative and population genomic landscape of *Phellinus noxius*: A hypervariable fungus causing root rot in trees. Mol. Ecol. 26(22):6301–6316. https://doi.org/10.1111/mec.14359.

Garcia JF, Figueroa-Balderas R, Comont G, Delmas CEL, Baumgartner K, Cantu D. 2024. Genome analysis of the esca-associated Basidiomycetes *Fomitiporia mediterranea*, *Fomitiporia polymorpha*, *Inonotus vitis*, and *Tropicoporus texanus* reveals virulence factor repertoires characteristic of white-rot fungi. G3-Genes Genomes Genet. 14(10). https://doi.org/10.1093/g3journal/jkae189.

Hao JH, Wang XL, Shi YH, Li LJ, Chu JX, Li JJ, Lin WP, Yu T, Hou AH. 2023. Integrated omic profiling of the medicinal mushroom *Inonotus obliquus* under submerged conditions. BMC Genomics. 24(1):554. https://doi.org/10.1186/s12864-023-09656-z.

Hage H, Miyauchi S, Virágh M, Drula E, Min B, Chaduli D, Navarro D, Favel A, Norest M, Lesage-Meessen L, et al. 2021. Gene family expansions and transcriptome signatures uncover fungal adaptations to wood decay. Environ. Microbiol. 23(10):5716-5732. https://doi.org/10.1111/1462-2920.15423.

Jin C, Ma JX, Wang H, Tang LX, Ye YF, Li X, Si J. 2024. First genome assembly and annotation of *Sanghuangporus weigelae* uncovers its medicinal functions, metabolic pathways, and evolution. Front. Cell. Infect. Microbiol. 13:1325418. https://doi.org/10.3389/fcimb.2023.1325418.

Ma JX, Wang H, Jin C, Ye YF, Tang LX, Si J, Song J. 2024. Whole genome sequencing and annotation of *Daedaleopsis sinensis*, a wood-decaying fungus significantly degrading lignocellulose. Front. Bioeng. Biotechnol. 11:1325088. https://doi.org/10.3389/fbioe.2023.1325088.

Ma XX, Lu LX, Yao FJ, Fang M, Wang P, Meng JJ, Shao KS, Sun X, Zhang YM. 2023. High-quality genome assembly and multi-omics analysis of pigment synthesis pathway in *Auricularia cornea.* Front Microbiol. 14:1211795. https://doi.org/10.3389/fmicb.2023.1211795.

Wang SX, Liu ZC, Wang XT, Liu RP, Zou L. 2022. Mushrooms Do poduce favonoids: metabolite profiling and transcriptome analysis of flavonoid synthesis in the medicinal mushroom *Sanghuangporus baumii*. J. Fungi. 8(6):582. https://doi.org/10.3390/jof8060582.

Wei JS, Liu LY, Yuan XL, Wang D, Wang XY, Bi W, Yang Y, Wang Y. 2023. Transcriptome analysis reveals the putative polyketide synthase gene involved in hispidin biosynthesis in *Sanghuangporus sanghuang.* Mycobiology. 51(5):360-371. https://doi.org/10.1080/12298093.2023.2257999.

Zhu YJ, Xu J, Sun C, Zhou SG, Xu HB, Nelson DR, Qian J, Song JY, Luo HM, Li Xiang L, et al. 2015. Chromosome-level genome map provides insights into diverse defense mechanisms in the medicinal fungus *Ganoderma sinense.* Sci. Rep. 5:11087. https://doi.org/10.1038/srep11087.
